# Plasma miR-148b-3p is associated with CKD-related renal dysfunction and carotid plaque burden in patients with established coronary artery disease

**DOI:** 10.3389/fcvm.2026.1908726

**Published:** 2026-08-12

**Authors:** Wenbo Yang, Heze Fan, Wenjiao Zhang, Yirui Xu, Qi Kang, Shumei Zhang, Xueying Feng, Erwei Hu, Peiyao Zhang, Jiandong Wang, Hao Yuan, Lijun Wang, Zuyi Yuan, Juan Zhou

**Affiliations:** 1 Department of Cardiology, The First Affiliated Hospital of Xi’an Jiaotong University, Xi’an, Shaanxi, China; 2 Key Laboratory of Environment and Genes Related to Diseases, Ministry of Education, Xi’an, China; 3 Department of Cardiology, The People’s Hospital of Jingbian, Jingbian County, Shaanxi, China

**Keywords:** carotid atherosclerosis, chronic kidney disease, coronary artery disease, endothelial dysfunction, miR-148b-3p

## Abstract

**Background:**

Chronic kidney disease (CKD) is associated with increased atherosclerotic burden and vascular risk, particularly in patients with established coronary artery disease (CAD). Circulating microRNAs may reflect renal dysfunction and systemic vascular injury. We investigated whether plasma miR-148b-3p is associated with renal function and carotid plaque burden in patients with established CAD.

**Methods:**

We conducted a single-center cross-sectional study of 190 hospitalized patients with coronary artery disease, including 90 with CKD and 100 without CKD. Plasma miR-148b-3p was measured by quantitative polymerase chain reaction. Carotid atherosclerosis was assessed by ultrasonography and quantified by Crouse score. Associations with renal function and plaque burden were evaluated using correlation analysis, multivariable regression, restricted cubic spline analysis, ROC analysis, bootstrap validation, and incremental predictive analyses. Bioinformatic and transcriptomic analyses were performed to identify hypothesis-generating candidate targets.

**Results:**

In patients with established CAD, plasma miR-148b-3p levels were higher in CKD than non-CKD patients [8.77 (7.35, 11.08) vs. 2.43 (1.41, 3.45), *P* = 7.497 × 10^−5^]. Higher miR-148b-3p was independently associated with lower eGFR (standardized *β* = −0.306, 95% CI −0.441 to −0.170, *P* = 1.455 × 10^−5^) and greater Crouse score (*β* = 0.160, 95% CI 0.028–0.292, *P* = 0.017). For carotid plaque detection, in exploratory ROC analyses, plasma miR-148b-3p showed limited discrimination in non-CKD patients and moderate discrimination in the CKD subgroup. Although bootstrap resampling suggested internal stability, these estimates require external validation. Integrative analyses identified enrichment of endothelial and atherogenic pathways and highlighted 4 shared candidate genes—MRPS25, ADAMTS5, MMP15, and RAB12.

**Conclusions:**

In patients with established CAD, plasma miR-148b-3p was associated with CKD status, impaired renal function, and carotid plaque burden. Its potential adjunctive value for plaque assessment remains exploratory and requires confirmation in larger prospective cohorts with independent external validation.

## 1 Introduction

Cardiovascular-kidney-metabolic (CKM) syndrome is a clinical syndrome in which metabolic risk factors, chronic kidney disease (CKD), and cardiovascular disease coexist and mutually reinforce one another (1). Within this framework, CKD is both a major component of CKM and a strong determinant of adverse cardiovascular outcomes (1, 2). Patients with CKD have a substantially higher burden of atherosclerotic cardiovascular disease than the general population, and this risk increases progressively with worsening renal function (2, 3). Notably, the excess cardiovascular risk associated with CKD is not fully captured by conventional risk factors alone (3). Among the vascular manifestations of CKD, carotid atherosclerosis is particularly relevant because it provides a noninvasive marker of systemic vascular injury and independently predicts future cardiovascular events (4).

MicroRNAs (miRNAs) have emerged as key post-transcriptional regulators linking renal dysfunction to vascular remodeling within the CKM spectrum (5, 6). By modulating inflammatory signaling, oxidative stress, fibrosis, and endothelial homeostasis, miRNAs participate in both CKD progression and atherogenesis (7). Increasing evidence suggests that circulating miRNAs are attractive biomarkers because of their relative stability and their ability to reflect ongoing intercellular and systemic pathophysiologic processes (8). Among the vascular mechanisms involved, endothelial dysfunction is especially relevant in CKD-mediated atherosclerosis, as it represents an early and central event that promotes leukocyte recruitment, lipid deposition, and plaque development (3, 9).

MiR-148b-3p has attracted growing interest because its reported functions intersect renal injury, inflammation, and endothelial biology (10, 11). Prior studies suggest that miR-148b-3p has been reported in studies related to endothelial activation, angiogenic regulation, and injury-associated signaling (11, 12). Emerging evidence has linked miR-148b to kidney disease progression and metabolic dysregulation, suggesting that it may represent a molecular node at the intersection of renal injury and vascular risk (13, 14). However, its role in CKD-associated atherosclerosis remains poorly defined. Based on these considerations, we investigated whether plasma miR-148b-3p is associated with renal function and carotid plaque burden in patients with established CAD, with particular emphasis on differences between patients with and without CKD.

## 2 Methods

### 2.1 Study design and participants

This was a single-center, cross-sectional study conducted at the First Affiliated Hospital of Xi'an Jiaotong University. A total of 190 hospitalized patients with CAD between 2020 and 2021 were enrolled, including 90 patients with CKD and 100 patients without CKD. Demographic characteristics, medical history, medication use, admission laboratory data, and carotid ultrasonographic findings were collected for all participants. Peripheral blood samples were obtained for measurement of plasma miR-148b-3p. The study was designed to evaluate the associations of plasma miR-148b-3p with renal function, carotid plaque burden, and the predictive value of miR-148b-3p for carotid atherosclerosis in patients with and without CKD.

### 2.2 Inclusion and exclusion criteria

Patients were eligible if they met all of the following criteria: (1) CAD confirmed on the basis of clinical symptoms, electrocardiography, myocardial injury biomarkers, and coronary angiography; (2) age between 18 and 80 years; (3) availability of complete clinical data, including medical history, laboratory measurements, imaging examinations, and treatment records; and (4) relatively stable clinical status at enrollment, without acute myocardial infarction, severe arrhythmia, acute heart failure, or acute exacerbation of CKD.

Patients were excluded if they had any of the following: (1) acute coronary syndrome within the preceding 3 months; (2) severe comorbid conditions, including advanced liver failure, metastatic malignancy or malignancy receiving chemotherapy or radiotherapy, active autoimmune disease, or severe infection; (3) organ transplantation within the preceding 6 months or recent treatment with immunosuppressive agents or high-dose glucocorticoids; (4) severe psychiatric disease or cognitive impairment precluding reliable participation; or (5) pregnancy or lactation.

### 2.3 Diagnostic definitions

#### 2.3.1 Diagnosis of CKD

CKD was diagnosed according to the KDIGO 2024 Clinical Practice Guideline and was defined as abnormalities of kidney structure or function present for at least 3 months, with implications for health. CKD was considered present if either of the following criteria was met: (1) markers of kidney damage, including albuminuria, urine sediment abnormalities, persistent hematuria, electrolyte or other abnormalities due to tubular disorders, structural abnormalities detected by imaging, or histopathologic abnormalities; or (2) persistent reduction in kidney function, defined as eGFR <60 mL/min/1.73 m^2^ for ≥3 months after exclusion of reversible causes such as acute kidney injury. eGFR was calculated using the Chinese modified MDRD equation: eGFR = 175 × Scr^−1.234^ × age^−0.179^ × 0.79 if female, where Scr represents serum creatinine expressed in mg/dL. Serum creatinine values measured in μmol/L were converted to mg/dL by dividing by 88.4 before calculation.

#### 2.3.2 Diagnosis and scoring of carotid atherosclerosis

Carotid atherosclerosis was assessed by carotid ultrasonography according to the American Society of Echocardiography recommendations for carotid arterial plaque assessment by ultrasound. Carotid plaque was defined as either (1) any focal thickening of the arterial wall thought to be atherosclerotic in origin and encroaching into the lumen of any carotid segment, or (2) diffuse vessel wall atherosclerosis with CIMT ≥1.5 mm in any carotid segment. The guideline further recommends visual scanning for protuberant plaque first and, if absent, CIMT measurement to identify diffuse plaque.

Carotid atherosclerosis was assessed by standardized bilateral carotid ultrasonography. The common carotid artery, carotid bifurcation, internal carotid artery, and external carotid artery were systematically examined. Carotid plaque was defined as a focal structure encroaching into the arterial lumen with a thickness ≥1.5 mm or a focal wall thickening ≥50% greater than the adjacent intima-media thickness.

Plaque burden was quantified using the conventional Crouse score. For each plaque, the maximal plaque thickness was measured in millimeters, and the Crouse score was calculated as the sum of maximal plaque thicknesses across all examined carotid segments.

Plaque echogenicity, surface morphology, and contrast-enhanced ultrasonography-derived intraplaque neovascularization were assessed as descriptive plaque characteristics only and were not included in the numerical Crouse score. Ultrasound measurements were performed by experienced sonographers who were blinded to CKD status and plasma miR-148b-3p levels.

### 2.4 Blood collection and plasma processing

Peripheral venous blood was collected into EDTA anticoagulant tubes according to a standardized protocol. In line with published methodological recommendations for circulating plasma miRNA analysis, plasma was separated by centrifugation and further processed to obtain platelet-poor plasma, thereby minimizing cellular contamination before RNA extraction. Plasma aliquots were labeled and stored at −80 °C until analysis.

### 2.5 Plasma miRNA extraction and quantitative PCR for miR-148b-3p

Total RNA, including small RNA, was isolated from 500 μL of plasma using TRIzol LS reagent (Invitrogen, USA) according to the manufacturer's instructions. Briefly, plasma samples were thawed on ice and mixed thoroughly with 1.5 mL TRIzol LS reagent. After sample lysis and before RNA extraction, synthetic cel-miR-39-3p spike-in control was added to each sample at 5.6 × 10^8^ copies, corresponding to 3.5 μL of a 1.6 × 10^8^ copies/μL working solution, as an exogenous spike-in control to monitor RNA extraction efficiency and reverse-transcription variability. RNA was extracted according to the manufacturer's protocol and eluted in RNase-free water.

RNA quantity and purity were assessed using a NanoDrop spectrophotometer by measuring absorbance at 260 and 280 nm. Reverse transcription was performed using the TaqMan MicroRNA Reverse Transcription Kit (Thermo Fisher Scientific, USA) together with miRNA-specific stem-loop reverse-transcription primers for miR-148b-3p and cel-miR-39-3p. Complementary DNA was synthesized from 10 ng of total RNA according to the manufacturer's protocol.

Quantitative real-time PCR was performed using GoTaq qPCR Master Mix (Promega, USA), a dye-based quantitative PCR chemistry, together with sequence-specific forward primers and a universal reverse primer. Each sample was analyzed in technical triplicate reactions, and the mean Ct value was used for subsequent analysis. Replicates with a Ct difference greater than 0.5 cycles were repeated or excluded. No-template controls and no-reverse-transcription controls were included to monitor contamination and nonspecific amplification. All samples included in the final analytic cohort passed the predefined qPCR quality-control criteria.

Because dye-based qPCR chemistry was used, amplification specificity was assessed by melting curve analysis. Amplification efficiency was evaluated using standard curves generated from serially diluted cDNA. Assays with amplification efficiencies within 90%–110% and correlation coefficients *R*^2^ ≥ 0.99 were considered acceptable. Relative plasma miR-148b-3p expression was calculated using the 2^−ΔΔCt^ method and normalized to cel-miR-39-3p. Primer sequences used in this study are provided in Supplementary Table S1.

### 2.6 Clinical data collection and subgroup stratification

Baseline clinical data included age, sex, body mass index, smoking history, prior stroke, prior percutaneous coronary intervention, medication use, and cardiovascular risk factors such as hypertension and diabetes mellitus. Admission laboratory measurements included lipid parameters and renal function indices. These clinical variables were used for baseline comparisons and multivariable adjustment in regression analyses.

For subgroup analyses, patients were stratified according to plasma miR-148b-3p levels into 3 tertiles, defined as the low-, middle-, and high-expression groups. Clinical characteristics, plaque-related risk factors, and Crouse scores were then compared across the tertile groups.

### 2.7 Bioinformatic prediction of miR-148b-3p target genes

Putative target genes of miR-148b-3p were predicted using the miRWalk (http://mirwalk.umm.uniheidelberg.de/) and TargetScan (https://www.targetscan.org/) databases. MiRWalk provides predicted and validated miRNA–target interactions, whereas TargetScan predicts biological targets on the basis of complementary seed-sequence matching and sequence context features. Candidate downstream genes supported by both databases were selected for subsequent functional annotation and integrative analyses.

### 2.8 GO and KEGG enrichment analyses

Functional enrichment analysis of candidate miR-148b-3p target genes was performed using the Database for Annotation, Visualization and Integrated Discovery (DAVID). Gene lists were uploaded into the DAVID Functional Annotation Tool, and Gene Ontology (GO) and Kyoto Encyclopedia of Genes and Genomes (KEGG) pathway analyses were performed to identify enriched biological processes and signaling pathways relevant to endothelial biology and atherosclerosis.

### 2.9 Single-cell RNA-sequencing analysis of GSE253903

Public single-cell RNA-sequencing data from GSE253903 were analyzed in R using the Seurat package. The dataset included 12 human carotid plaque samples, comprising 6 asymptomatic and 6 symptomatic specimens. Raw count matrices were imported to generate Seurat objects for each sample, followed by data integration and quality control to remove low-quality cells and cells with excessive mitochondrial transcript content. After normalization, highly variable genes were identified, and the data were scaled for downstream dimensionality reduction. Principal component analysis was performed, and graph-based clustering was subsequently used to define cell populations, which were visualized by Uniform Manifold Approximation and Projection (UMAP). Endothelial cell clusters were identified according to canonical endothelial markers, including PECAM1, CDH5, VWF, CLDN5, ERG, KDR, and FLT1. Differential expression analysis was then restricted to the endothelial compartment to compare symptomatic and asymptomatic plaques and to identify endothelial genes downregulated in symptomatic lesions.

### 2.10 CKD endothelial transcriptomic reference dataset and integrative analysis

To prioritize biologically relevant downstream targets of miR-148b-3p in CKD-associated endothelial dysfunction, downregulated endothelial genes from GSE253903 were integrated with downregulated genes from our previously CKD endothelial transcriptomic dataset and with predicted miR-148b-3p target genes. In the reference CKD endothelial transcriptomic dataset, CKD was induced in C57BL/6J mice by 5/6 nephrectomy combined with a high-fat diet and tail-vein injection of PCSK9, whereas sham-operated mice served as controls. Aortic endothelial cells were isolated from CKD and sham mice and subjected to RNA sequencing (15). Differentially expressed genes were defined using an adjusted *P* value ≤0.05 and an absolute log2 fold change ≥0.5, and genes shared across datasets were considered high-priority candidate targets for downstream biological interpretation.

### 2.11 Statistical analysis

Statistical analyses were performed using GraphPad Prism 8.0. All variables included in the primary analyses, including demographic characteristics, cardiovascular risk factors, medication use, renal function indices, lipid parameters, plasma miR-148b-3p levels, carotid plaque status, and Crouse score, were complete for the final analytic cohort. Therefore, no imputation was performed. Categorical variables were expressed as number (percentage) and compared using the *χ*^2^ test. Continuous variables were expressed as median (interquartile range), continuous variables with normal distribution were compared using Student's *t* test or one-way ANOVA, whereas non-normally distributed variables were compared using nonparametric tests. Associations between plasma miR-148b-3p and clinical variables were assessed using Spearman correlation analysis. Associations between miR-148b-3p and carotid plaque burden were further evaluated using linear regression analysis. Three multivariable models were constructed. Model 1 was adjusted for age, sex, and body mass index. Model 2 was adjusted for the covariates in Model 1 plus cardiovascular risk factors, including hypertension, diabetes mellitus, smoking, and lipid parameters (total cholesterol, triglycerides, high-density lipoprotein cholesterol, and low-density lipoprotein cholesterol). Model 3 was adjusted for the covariates in Model 2 plus current medications, including ACEI, ARB, β-blockers, and statins. Restricted cubic spline (RCS) analysis was performed to assess potential nonlinear associations between eGFR and miR-148b-3p levels, as well as between miR-148b-3p levels and carotid plaque burden as measured by the Crouse score. Multicollinearity in fully adjusted models was assessed using variance inflation factors, with VIF values >5 considered suggestive of moderate collinearity and values >10 indicating substantial collinearity. The distribution of miR-148b-3p and the assumptions of the linear regression models were assessed using the Shapiro–Wilk, Breusch–Pagan, and Ramsey RESET tests.

Receiver operating characteristic (ROC) analyses were performed separately in the CKD and non-CKD groups to evaluate the discriminative performance of plasma miR-148b-3p for carotid plaque. Plasma miR-148b-3p was used as the test variable, and carotid plaque status was used as the state variable. The optimal cutoff was determined using the maximum Youden index, and the area under the curve (AUC), sensitivity, specificity, and 95% confidence interval were calculated. Exploratory ROC analyses were also performed for cystatin C (Cys-C) using the same outcome definition and subgroup structure to provide a descriptive comparison with an established cardiorenal biomarker. To assess the internal stability of the AUC estimates, bootstrap resampling with replacement was performed for both miR-148b-3p and Cys-C using 1,000 iterations. The AUC was recalculated in each bootstrap sample, and bootstrap mean AUCs and percentile-based 95% confidence intervals were obtained. Because of the modest subgroup sizes and imbalanced distribution of plaque status, comparisons between miR-148b-3p and Cys-C were considered descriptive and exploratory and were not interpreted as demonstrating biomarker superiority. To evaluate the incremental discriminative value of plasma miR-148b-3p beyond established clinical variables, nested logistic regression models were constructed for carotid plaque. Changes in AUC were assessed using the DeLong test, improvement in model fit was evaluated using the likelihood ratio test, and reclassification performance was assessed using continuous net reclassification improvement and integrated discrimination improvement. Bootstrap resampling with 1,000 iterations was used to estimate 95% confidence intervals for these incremental performance measures. All statistical tests were two-sided, and *P* < 0.05 was considered statistically significant. Improvement in model fit was evaluated using the likelihood ratio test. Reclassification performance was assessed using continuous net reclassification improvement (NRI) and integrated discrimination improvement (IDI). Continuous NRI was calculated without predefined risk categories and quantified the extent to which addition of miR-148b-3p moved predicted risks upward among patients with carotid plaque and downward among patients without carotid plaque. IDI was calculated as the difference in discrimination slopes between the extended model and the base clinical model. Bootstrap resampling with 1,000 iterations was used to estimate 95% confidence intervals for ΔAUC, continuous NRI, and IDI.

*Post-hoc* detectable-effect analyses were performed to evaluate whether the available sample size was adequate for renal-function-related analyses of plasma miR-148b-3p. These analyses were descriptive and were not used for prospective sample-size planning. For correlation analyses between miR-148b-3p and renal-function indices, including eGFR and Cys-C, achieved power and the approximate sample size required to reach 80% power were estimated using Fisher z-transformation based on the observed Spearman correlation coefficients, with a two-sided *α* of 0.05 (Supplementary Table S5).

### 2.12 Ethics statement

This study was approved by the local ethics committee of the First Affiliated Hospital of Xi'an Jiaotong University (XJTU1AF2016LSL-036; 2016/12/29) and was conducted in accordance with the principles of the Declaration of Helsinki. All participants provided written informed consent before enrollment.

## 3 Results

### 3.1 Baseline characteristics and carotid plaque burden by CKD status

A total of 190 patients with CAD were included in this study, comprising 90 patients with CKD and 100 without CKD. Compared with the non-CKD group, patients in the CKD group were more frequently male and had a higher prevalence of hypertension, diabetes mellitus, prior stroke, prior PCI, and ARB use; they also had significantly lower eGFR (all *P* < 0.05) (Table 1). Overall, these findings indicate that the CKD group had a less favorable baseline cardiovascular risk profile.

**Table 1:** Baseline characteristics of patients with coronary artery disease according to CKD status.

Variable	CKD group (*n* = 90)	non-CKD group (*n* = 100)	*P* value
Sex, *n* (%)			0.019
Male	66 (73.33)	57 (57.00)	
Female	24 (26.67)	43 (43.00)	
Age, years	71 (60, 78.25)	63 (55, 70)	2.265 × 10^−4^
BMI, kg/m^2^	25.61 (23.08, 27.74)	25.35 (23.00, 27.54)	0.854
Smoking history, *n* (%)	31 (34.44)	40 (40.00)	0.429
Hypertension, *n* (%)	78 (86.67)	69 (69.00)	0.004
Diabetes mellitus, *n* (%)	38 (42.22)	27 (27.00)	0.027
Stroke, *n* (%)	25 (27.78)	13 (13.00)	0.011
PCI, *n* (%)	29 (32.22)	9 (9.00)	1.367 × 10^−4^
ACEI use, *n* (%)	12 (13.33)	8 (8.00)	0.232
ARB use, *n* (%)	39 (43.33)	25 (25.00)	0.008
β-blocker use, *n* (%)	35 (38.89)	22 (22.00)	0.011
Statin use, *n* (%)	37 (41.11)	34 (34.00)	0.312
TC, mmol/L	3.44 (2.75, 4.41)	3.66 (3.12, 4.55)	0.101
TG, mmol/L	1.02 (0.73, 1.57)	1.14 (0.79, 1.55)	0.541
HDL-C, mmol/L	0.91 (0.76, 1.17)	1.01 (0.87, 1.20)	0.057
LDL-C, mmol/L	1.86 (1.30, 2.49)	2.03 (1.53, 2.77)	0.105
Cys-C, mg/L	1.50 (1.16, 1.89)	0.85 (0.65, 1.08)	4.080 × 10^−19^
eGFR, mL/min/1.73 m^2^	65.55 (47.75, 84.52)	141.39 (123.72, 163.02)	1.406 × 10^−32^

Values are presented as median (interquartile range) for continuous variables and *n* (%) for categorical variables, unless otherwise indicated. Between-group comparisons were performed using the Mann–Whitney *U* test for continuous variables and the *χ*^2^ test or Fisher's exact test for categorical variables, as appropriate. ACEI, angiotensin-converting enzyme inhibitor; ARB, angiotensin receptor blocker; BMI, body mass index; CKD, chronic kidney disease; eGFR, estimated glomerular filtration rate; HDL-C, high-density lipoprotein cholesterol; LDL-C, low-density lipoprotein cholesterol; PCI, percutaneous coronary intervention; TC, total cholesterol; TG, triglycerides; Cys-C, cystatin C.

Carotid plaque burden was significantly greater in the CKD group than in the non-CKD group. The prevalence of carotid atherosclerotic plaque was 82.22% (74/90) in the CKD group vs. 46.00% (46/100) in the non-CKD group (*P* < 0.001). Plaque thickness was also significantly higher in the CKD group, with a median value of 2.60 (1.50, 4.63) mm, compared with 0 (0, 3.18) mm in the non-CKD group (*P* < 0.001) (Table 2). Taken together, these data support a substantially greater carotid plaque burden in patients with CKD.

**Table 2:** Carotid plaque burden according to CKD status.

Variable	CKD group (*n* = 90)	non-CKD group (*n* = 100)	*P* value
Carotid plaque, *n* (%)	74 (82.22)	46 (46.00)	2.52 × 10^−6^
Plaque thickness, mm	2.60 (1.50, 4.63)	0 (0, 3.18)	5.23 × 10^−7^

### 3.2 Plasma miR-148b-3p and renal function

Plasma miR-148b-3p levels were markedly elevated in patients with CKD. The median plasma miR-148b-3p level was 8.77 (7.35, 11.08) in the CKD group and 2.43 (1.41, 3.45) in the non-CKD group (*P* = 7.497 × 10^−5^) (Table 3). This finding supports a close association between plasma miR-148b-3p and CKD status.

**Table 3:** Plasma miR-148b-3p expression according to CKD status.

Group	Plasma miR-148b-3p level
CKD group (*n* = 90)	8.77 (7.35, 11.08)
non-CKD group (*n* = 100)	2.43 (1.41, 3.45)
*P* value	7.497 × 10^−5^

To further examine the clinical correlates of miR-148b-3p, the overall study population was stratified into low-, middle-, and high-expression groups according to tertiles of plasma miR-148b-3p. Significant between-group differences were observed for sex distribution, hypertension, diabetes mellitus, HDL-C, and eGFR (all *P* < 0.05), whereas age, BMI, smoking history, plaque prevalence, plaque thickness, use of ACEI/ARB/β-blockers/statins, and levels of TC, TG, and LDL-C did not differ significantly (Table 4). Higher miR-148b-3p expression was therefore associated with a less favorable clinical phenotype, particularly poorer renal function and greater cardiometabolic comorbidity.

**Table 4:** Clinical and carotid plaque characteristics across tertiles of plasma miR-148b-3p expression.

Variable	Low expression group (*n* = 64)	Moderate expression group (*n* = 61)	High expression group (*n* = 65)	*P* value
Sex, *n* (%)				0.012
Male	39 (60.94)	33 (54.10)	51 (78.46)	
Female	25 (39.06)	28 (45.90)	14 (21.54)	
Age, years	65 (52, 78)	66 (55, 77)	63 (51, 75)	0.529
BMI, kg/m^2^	25.08 (22.67, 26.60)	25.91 (23.35, 27.54)	25.71 (23.38, 28.08)	0.259
Smoking history, *n* (%)	20 (31.25)	23 (37.70)	28 (43.08)	0.381
Hypertension, *n* (%)	38 (59.38)	51 (83.61)	58 (89.23)	0.001
Diabetes mellitus, *n* (%)	15 (23.44)	21 (34.43)	29 (44.62)	0.040
Stroke, *n* (%)	16 (25.00)	12 (19.67)	10 (15.38)	0.393
PCI, *n* (%)	16 (25.00)	10 (16.39)	12 (18.46)	0.451
Carotid plaque, *n* (%)	37 (57.81)	37 (60.66)	46 (70.77)	0.277
Plaque thickness, mm	1.50 (0.00, 3.82)	1.90 (0.00, 3.80)	2.30 (0.00, 3.95)	0.318
ACEI use, *n* (%)	5 (7.81)	6 (9.84)	9 (13.85)	0.524
ARB use, *n* (%)	27 (42.19)	14 (22.95)	23 (35.38)	0.071
β-blocker use, *n* (%)	21 (32.81)	15 (24.59)	21 (32.31)	0.534
Statin use, *n* (%)	28 (43.75)	18 (29.51)	25 (38.46)	0.252
TC, mmol/L	3.62 (2.96, 4.29)	3.69 (3.13, 4.50)	3.36 (2.76, 4.54)	0.449
TG, mmol/L	1.10 (0.77, 1.45)	1.03 (0.78, 1.35)	1.06 (0.74, 1.76)	0.867
HDL-C, mmol/L	1.02 (0.86, 1.20)	1.02 (0.86, 1.23)	0.84 (0.71, 1.04)	8.870 × 10^−4^
LDL-C, mmol/L	1.89 (1.46, 2.54)	2.04 (1.46, 2.81)	1.98 (1.42, 2.52)	0.682
Cys-C, mg/L	1.07 (0.80, 1.30)	1.02 (0.79, 1.34)	1.36 (0.84, 1.83)	1.008 × 10^−2^
eGFR, mL/min/1.73 m^2^	116.06 (86.40, 143.89)	130.58 (74.44, 154.93)	73.58 (50.10, 122.63)	4.705 × 10^−5^

**Figure 1:**
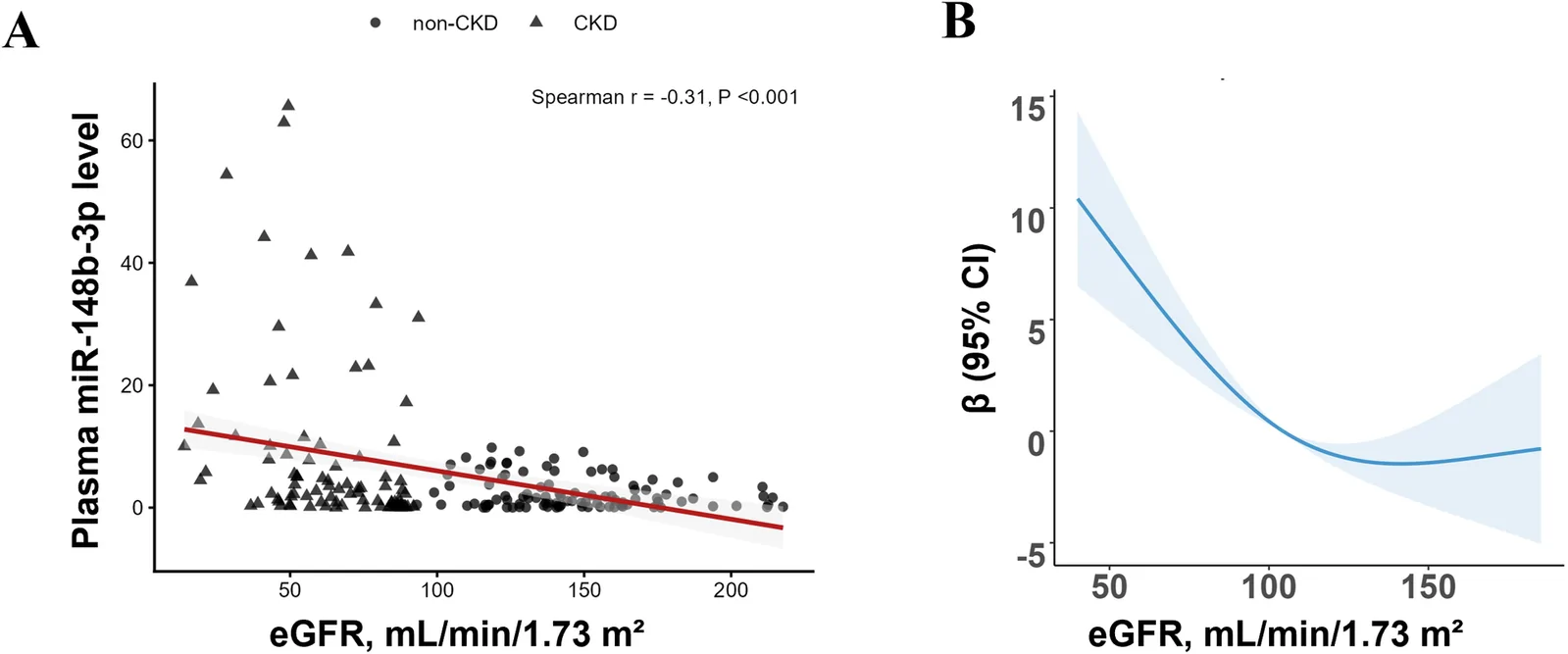
Plasma miR-148b-3p is inversely associated with renal function. **(A)** Spearman correlation analysis showing the association between plasma miR-148b-3p level and eGFR in the overall study population. **(B)** Restricted cubic spline analysis showing the nonlinear relationship between eGFR and plasma miR-148b-3p. The shaded area represents the 95% confidence interval. eGFR, estimated glomerular filtration rate.

Spearman correlation analysis showed a significant inverse association between plasma miR-148b-3p and eGFR (*r* = −0.310, *P* < 0.001), indicating that miR-148b-3p increased as renal function declined (Figure 1A). In addition, plasma miR-148b-3p was positively correlated with cystatin C (Cys-C) (Spearman *r* = 0.22, *P* = 0.002), providing further support for an association between higher miR-148b-3p levels and impaired renal function (Supplementary Figure S1). We further evaluated the association between plasma miR-148b-3p levels and eGFR using univariable and multivariable linear regression models. As shown in Table 5, higher plasma miR-148b-3p expression was significantly associated with lower eGFR in the unadjusted model (standardized *β* = −0.364, 95% CI −0.498 to −0.230, *P* = 2.508 × 10^−7^). This inverse association remained significant after adjustment for age, sex, and BMI in Model 1 (*β* = −0.325, 95% CI −0.458 to −0.192, *P* = 2.949 × 10^−6^). After further adjustment for hypertension, diabetes, smoking status, and lipid profiles, including TC, TG, HDL-C, and LDL-C, the association was slightly attenuated but remained significant in Model 2 (*β* = −0.307, 95% CI −0.441 to −0.172, *P* = 1.282 × 10^−5^). In the fully adjusted model, which additionally included current medication use, including ACE inhibitors, ARBs, β-blockers, and statins, plasma miR-148b-3p remained independently and inversely associated with eGFR (*β* = −0.306, 95% CI −0.441 to −0.170, *P* = 1.455 × 10^−5^) (Table 5). To evaluate the stability of the fully adjusted models, variance inflation factor (VIF) analysis was performed. In the eGFR Model 3, the VIF for miR-148b-3p was 1.13, indicating no meaningful multicollinearity between miR-148b-3p and the adjusted covariates. Most covariates had VIF values below 2.0, whereas TC and LDL-C showed moderate collinearity, with VIF values of 6.57 and 6.62, respectively. These results suggest that the independent association between miR-148b-3p and eGFR was not materially affected by multicollinearity (Supplementary Table S2). Restricted cubic spline analysis further supported a nonlinear association between eGFR and plasma miR-148b-3p levels, with higher miR-148b-3p levels observed as renal function declined (Figure 1B). Across the entire cohort, mean plasma miR-148b-3p levels increased from the G1 through G4 eGFR categories, with the highest level observed in G4. In category-specific analyses, plasma miR-148b-3p was inversely associated with eGFR only in the G2 category (Spearman *ρ* = −0.368; *P* = 0.008), suggesting that miR-148b-3p may be sensitive to early declines in kidney function. No significant associations were observed in the G1, G3, or G4 categories. Because only 6 participants were classified as G4 and 1 as G5, estimates in advanced kidney dysfunction were imprecise and should be interpreted cautiously (Supplementary Table S6). These findings support the potential value of circulating miR-148b-3p as a marker of renal functional impairment but require validation in larger, longitudinal cohorts. Taken together, these findings indicate that elevated plasma miR-148b-3p is closely associated with CKD status and impaired renal function.

**Table 5:** Association between plasma miR-148b-3p and renal function in linear regression analyses.

Model	Standardized coefficient *β* (95% CI)	*P* value
Unadjusted	−0.364 (−0.498, −0.230)	2.508 × 10^−7^
Model 1	−0.325 (−0.458, −0.192)	2.949 × 10^−6^
Model 2	−0.307 (−0.441, −0.172)	1.282 × 10^−5^
Model 3	−0.306 (−0.441, −0.170)	1.455 × 10^−5^

Standardized regression coefficients (*β*), 95% confidence intervals, and *P* values are shown for the association between plasma miR-148b-3p and eGFR. Model 1 was adjusted for age, sex, and BMI. Model 2 was adjusted for Model 1 covariates plus hypertension, diabetes mellitus, smoking, TC, TG, HDL-C, and LDL-C. Model 3 was adjusted for Model 2 covariates plus ACEI, ARB, β-blocker, and statin use.

### 3.3 Plasma miR-148b-3p and carotid atherosclerosis

To explore the association between plasma miR-148b-3p and plaque-related risk factors, Spearman correlation analysis was performed. Plasma miR-148b-3p was positively correlated with hypertension (*r* = 0.325, *P* = 0.020) and diabetes mellitus (*r* = 0.374, *P* = 0.012), but was not significantly associated with smoking history, TC, TG, HDL-C, or LDL-C (all *P* > 0.05) (Table 6). These findings suggest that higher miR-148b-3p expression was more closely associated with major cardiometabolic comorbidities than with conventional lipid parameters.

**Table 6:** Correlations between plasma miR-148b-3p and plaque-related cardiovascular risk factors.

Risk factor	Correlation coefficient (*r*)	*P* value
Hypertension	0.325	0.020
Diabetes mellitus	0.374	0.012
Smoking	0.175	0.076
TC	0.134	0.120
TG	0.124	0.095
HDL-C	−0.105	0.102
LDL-C	0.133	0.120

Carotid plaque burden, assessed by Crouse score, increased progressively across miR-148b-3p tertiles. The median Crouse score was 1.55 (0.00, 1.93) in the low-expression group, 2.40 (1.74, 3.15) in the moderate-expression group, and 3.20 (2.81, 4.80) in the high-expression group (*P* = 1.303 × 10^−4^). The high-expression group had a significantly higher Crouse score than both the middle- and low-expression groups, and the middle-expression group also had a significantly higher score than the low-expression group (all *P* < 0.05) (Table 7). These results indicate a stepwise positive association between plasma miR-148b-3p expression and carotid plaque burden.

**Table 7:** Crouse score across tertiles of plasma miR-148b-3p expression.

Group	Crouse score
Low miR-148b-3p expression group (*n* = 64)	1.55 (0.00, 1.93)
Moderate miR-148b-3p expression group (*n* = 61)	2.40 (1.74, 3.15)*
High miR-148b-3p expression group (*n* = 65)	3.20 (2.81, 4.80)*^,^**
*P* value	1.303 × 10^−4^

Values are presented as median (interquartile range). The overall study population was stratified into tertiles according to plasma miR-148b-3p expression. Overall between-group comparison was performed using the Kruskal–Wallis test. Pairwise comparisons were performed after the overall test.

* *P* < 0.05 vs. the low-expression group.

** *P* < 0.05 vs. the moderate-expression group.

**Figure 2:**
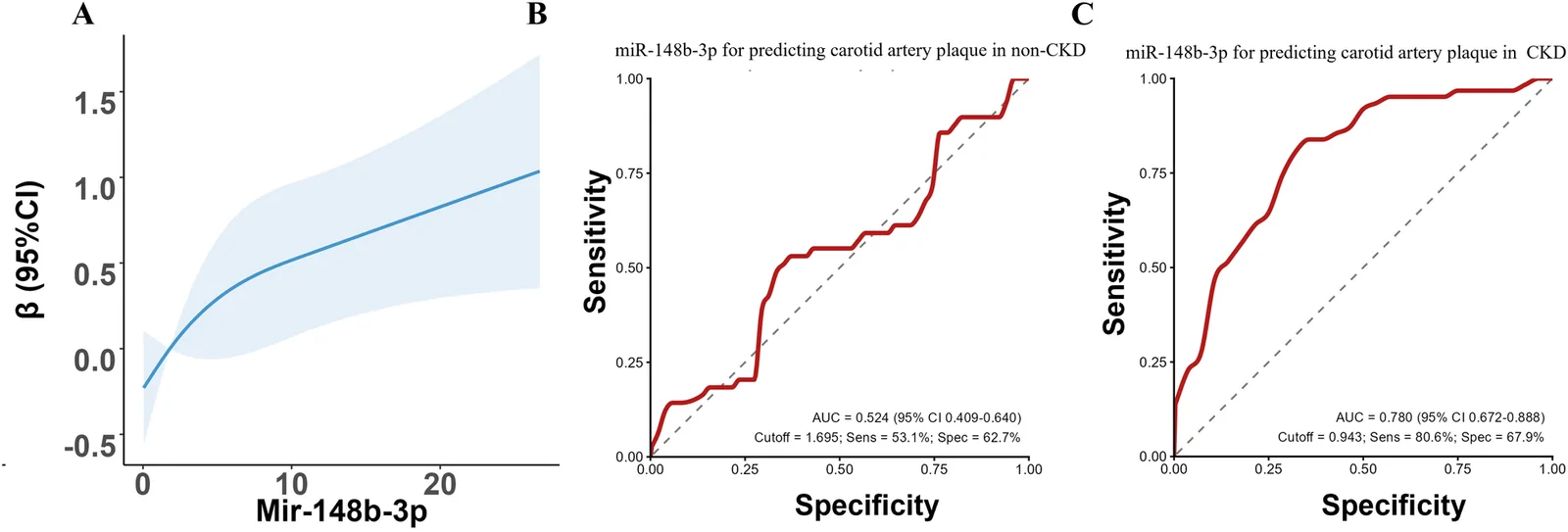
Association between plasma miR-148b-3p and carotid plaque burden in patients with established CAD. **(A)** Restricted cubic spline analysis showing the nonlinear positive association between plasma miR-148b-3p and Crouse score. The shaded area represents the 95% confidence interval. **(B)** Receiver operating characteristic curve for plasma miR-148b-3p in detecting carotid plaque in non-CKD patients (AUC = 0.524; 95% CI, 0.409–0.640; optimal cutoff = 1.695; sensitivity = 53.1%; specificity = 62.7%). **(C)** Receiver operating characteristic curve for plasma miR-148b-3p in detecting carotid plaque in CKD patients (AUC = 0.780; 95% CI, 0.672–0.888; optimal cutoff = 0.943; sensitivity = 80.6%; specificity = 67.9%).

Linear regression analysis further demonstrated a positive association between plasma miR-148b-3p and Crouse score. In the unadjusted model, miR-148b-3p was significantly associated with Crouse score (standardized *β* = 0.238, 95% CI 0.099–0.378, *P* = 0.001). This association remained significant after adjustment for age, sex, and BMI in Model 1 (*β* = 0.202, 95% CI 0.071–0.332, *P* = 0.003), and after further adjustment for hypertension, diabetes mellitus, smoking, TC, TG, HDL-C, and LDL-C in Model 2 (*β* = 0.164, 95% CI 0.034–0.295, *P* = 0.014). In the fully adjusted Model 3, which additionally included ACEI, ARB, β-blocker, and statin use, plasma miR-148b-3p remained independently associated with Crouse score (*β* = 0.160, 95% CI 0.028–0.292, *P* = 0.017) (Table 8). Restricted cubic spline analysis also suggested a positive exposure-response relationship between plasma miR-148b-3p and carotid plaque burden (Figure 2A).

**Table 8:** Association between plasma miR-148b-3p and carotid plaque burden in linear regression analyses.

Model	Standardized coefficient *β* (95% CI)	*P* value
Unadjusted	0.238 (0.099, 0.378)	0.001
Model 1	0.202 (0.071, 0.332)	0.003
Model 2	0.164 (0.034, 0.295)	0.014
Model 3	0.160 (0.028, 0.292)	0.017

Standardized regression coefficients (*β*), 95% confidence intervals, and *P* values are shown for the association between plasma miR-148b-3p and Crouse score. Model 1 was adjusted for age, sex, and BMI. Model 2 was adjusted for Model 1 covariates plus hypertension, diabetes mellitus, smoking, TC, TG, HDL-C, and LDL-C. Model 3 was adjusted for Model 2 covariates plus ACEI, ARB, β-blocker, and statin use.

Receiver operating characteristic analysis was used to assess the discriminative value of plasma miR-148b-3p for carotid plaque in patients with and without CKD. In exploratory ROC analyses, plasma miR-148b-3p showed limited discrimination for carotid plaque in the non-CKD group, with an AUC of 0.524 (95% CI 0.409–0.640). At the optimal cutoff value of 1.695, sensitivity was 53.1% and specificity was 62.7% (Figure 2B). In the CKD group, miR-148b-3p showed stronger discriminative performance, with an AUC of 0.780 (95% CI 0.672–0.888). At the optimal cutoff value of 0.943, sensitivity was 80.6% and specificity was 67.9% (Figure 2C). Bootstrap resampling yielded a mean AUC of 0.782 and a 95% CI of 0.667–0.885 in the CKD subgroup. However, given the modest subgroup size and the limited number of patients without carotid plaque, these results should be considered exploratory rather than evidence of established diagnostic performance (Supplementary Table S3). Addition of miR-148b-3p to the clinical model increased the AUC from 0.696 to 0.794, corresponding to a ΔAUC of 0.098. This improvement was significant by DeLong test (*P* = 0.019) and likelihood ratio test (*P* = 0.001). Continuous NRI was 0.682 and IDI was 0.114. Bootstrap analysis further supported the incremental value of miR-148b-3p in the CKD subgroup, with a bootstrap mean continuous NRI of 0.818 (95% CI, 0.088–1.615) and a bootstrap mean IDI of 0.141 (95% CI, 0.008–0.449) (Supplementary Table S4). Cys-C showed limited discrimination for carotid plaque in the non-CKD subgroup (AUC, 0.554; bootstrap 95% CI, 0.441–0.661) and modest discrimination in the CKD subgroup (AUC, 0.638; bootstrap 95% CI, 0.483–0.781). In the CKD subgroup, the apparent AUC was numerically higher for miR-148b-3p than for Cys-C (0.780 vs. 0.638). However, these exploratory estimates were derived from the same modest subgroup and should not be interpreted as demonstrating superiority or established diagnostic utility (Supplementary Table S10; Supplementary Figure S2). These results suggest that plasma miR-148b-3p had greater discriminative value for carotid plaque in patients with CKD than in those without CKD.

### 3.4 Predicted target landscape of miR-148b-3p and integrative endothelial transcriptomic analyses

**Figure 3:**
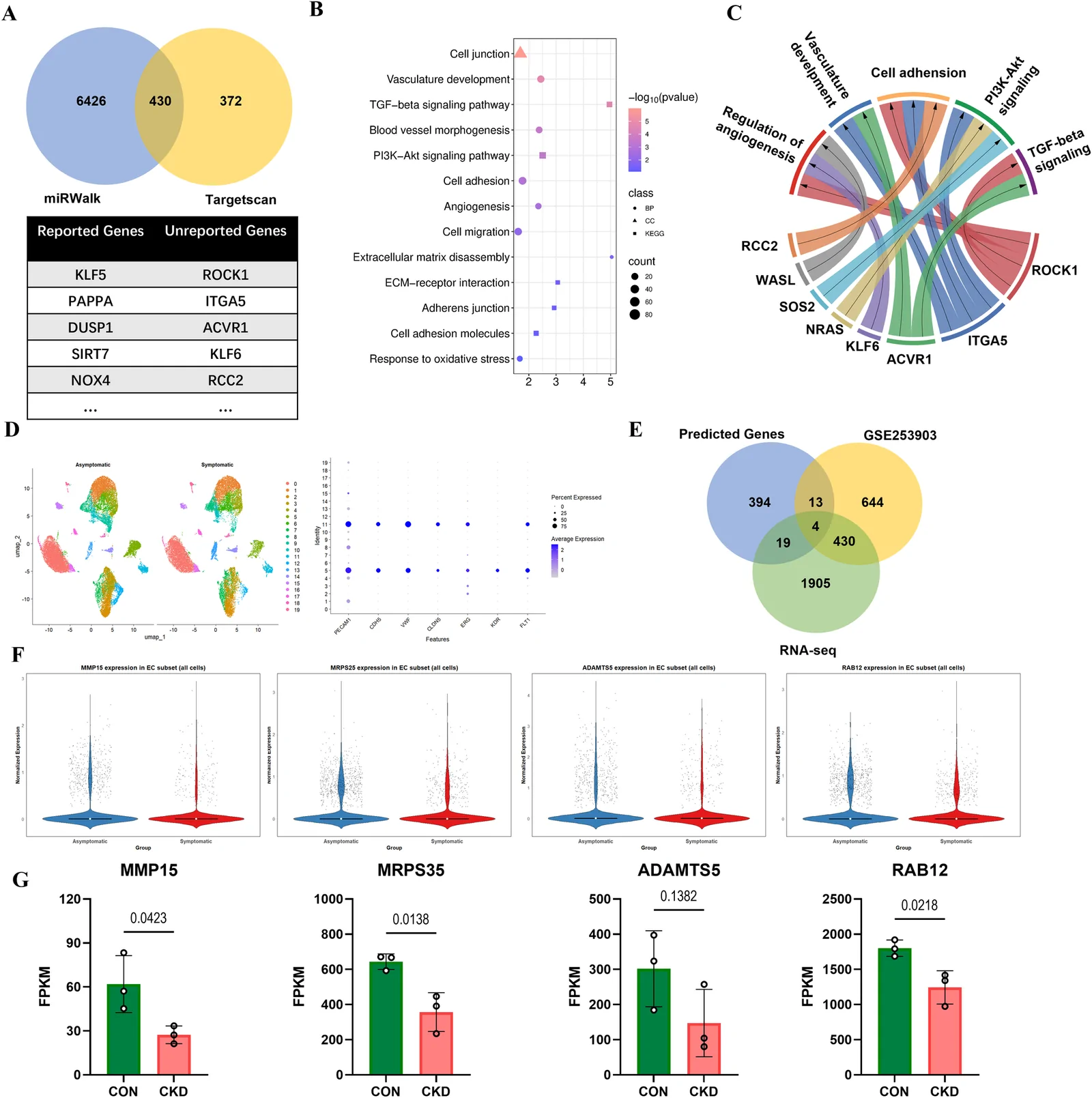
Predicted miR-148b-3p target landscape and integrative endothelial transcriptomic analysis. **(A)** Overlap of predicted miR-148b-3p target genes identified by miRWalk and TargetScan, yielding 430 shared candidate targets. **(B)** Functional enrichment analysis of shared target genes showing pathways related to endothelial biology and atherosclerosis, including angiogenesis, vasculature development, cell adhesion, PI3K-Akt signaling, and TGF-β signaling. **(C)** Chord diagram illustrating representative pathway-related candidate targets and their connections with endothelial and atherogenic pathways. **(D)** Single-cell RNA-Sequencing analysis of human carotid plaque dataset GSE253903, showing cell clustering and endothelial marker expression. **(E)** Integrative intersection of predicted miR-148b-3p targets, downregulated endothelial genes from GSE253903, and downregulated genes from the CKD endothelial RNA-Seq dataset. **(F)** Expression patterns of MRPS25, ADAMTS5, MMP15, and RAB12 in endothelial cells from GSE253903. **(G)** Expression changes of these four genes in the CKD endothelial RNA-Seq dataset.

To further define the downstream regulatory landscape of miR-148b-3p, target genes were predicted using 2 independent databases. MiRWalk identified 6865 candidate targets, whereas TargetScan identified 802 candidate targets. Intersection analysis yielded 430 shared target genes, which were subsequently subjected to GO and KEGG enrichment analyses (Figure 3A). These shared targets were significantly enriched in pathways relevant to atherosclerosis and endothelial biology, including regulation of angiogenesis, vasculature development, cell adhesion, PI3K-Akt signaling, and TGF-β signaling (Figure 3B).

Among these pathway-related genes, several reported or biologically plausible miR-148b-3p targets with vascular relevance were highlighted, including KLF5, PAPPA, DUSP1, SIRT7, and NOX4. Additional candidate genes enriched in these pathways included ROCK1, ITGA5, ACVR1, KLF6, and RCC2. A chord diagram further demonstrated broad connectivity between representative genes and endothelial or atherogenic pathways, with ROCK1, ITGA5, and ACVR1 showing links to multiple pathways (Figure 3C).

To further examine the endothelial relevance of these findings at single-cell resolution, GSE253903, a public single-cell RNA-sequencing dataset generated from human carotid endarterectomy specimens including asymptomatic and symptomatic plaques, was analyzed by single-cell RNA sequencing. UMAP visualization demonstrated clear cellular clustering, with broadly comparable distribution of major cell populations between the asymptomatic and symptomatic groups. Endothelial cell clusters were identified on the basis of canonical endothelial markers, including PECAM1, CDH5, VWF, CLDN5, ERG, KDR, and FLT1 (Figure 3D). Differential expression analysis restricted to endothelial cells showed that multiple endothelial-associated genes were decreased in the symptomatic group relative to the asymptomatic group, indicating loss of endothelial homeostatic transcriptional programs in clinically unstable plaques.

To prioritize candidate downstream effectors of miR-148b-3p in CKD-associated endothelial dysfunction, an integrative intersection analysis was performed using 3 datasets: downregulated endothelial differentially expressed genes from GSE253903, downregulated genes from our CKD endothelial sequencing dataset, and predicted miR-148b-3p target genes. This analysis identified 4 genes shared across all 3 datasets: MRPS25, ADAMTS5, MMP15, and RAB12 (Figures 3E–G). These genes are functionally related to mitochondrial homeostasis, extracellular matrix remodeling, autophagic-lysosomal trafficking, and angiogenic regulation. Together, these integrative analyses suggest that miR-148b-3p may be linked to endothelial dysfunction and plaque burden through coordinated effects on vascular remodeling, matrix regulation, and endothelial metabolic adaptation.

## 4 Discussion

The principal findings of this study are as follows. First, in patients with established CAD, those with CKD had a substantially greater carotid atherosclerotic burden, reflected by higher plaque prevalence, greater plaque thickness, and higher Crouse score. Second, plasma miR-148b-3p levels were significantly elevated in patients with CKD and were consistently associated with impaired renal function, as indicated by lower eGFR and higher Cys-C levels. Third, higher plasma miR-148b-3p levels were associated with greater carotid plaque burden and remained independently associated with Crouse score after adjustment for demographic factors, cardiovascular risk factors, lipid parameters, and medication use. Fourth, exploratory ROC analyses suggested limited discrimination for carotid plaque in the non-CKD group and moderate discrimination in the CKD subgroup. However, given the modest subgroup size, imbalanced plaque-status distribution, and absence of external validation, these findings should not be interpreted as establishing diagnostic utility. Finally, integrative bioinformatic, single-cell transcriptomic, and endothelial RNA-sequencing analyses identified several biologically plausible candidate targets that may be related to endothelial homeostasis, extracellular matrix remodeling, mitochondrial function, and autophagic-lysosomal pathways.

An important feature of the present study is its clinical design framework. This study did not aim to examine miR-148b-3p in relation to CAD itself, because all enrolled participants had established CAD. Instead, the primary objective was to determine whether, in patients with established CAD, CKD was associated with higher circulating miR-148b-3p levels and greater carotid atherosclerotic burden. This design allowed us to evaluate CKD-related vascular differences within a clinically relevant high-risk population. Such a framework is consistent with the CKM concept, in which renal dysfunction, metabolic abnormalities, and atherosclerotic cardiovascular disease frequently coexist and mutually reinforce risk. In this setting, the higher miR-148b-3p levels and greater carotid plaque burden observed in CKD patients suggest that plasma miR-148b-3p may reflect a CKD-associated vascular and metabolic milieu superimposed on established ASCVD.

Progressive CKD is accompanied by broad remodeling of the circulating miRNA landscape, and accumulating evidence supports circulating miRNAs as potential biomarkers of kidney injury, kidney function decline, and cardio-renal risk (7). Within this broader context, miR-148b appears biologically relevant. Previous studies have suggested that circulating miR-148b may distinguish certain primary glomerular diseases from controls, and renal miR-148b has been linked to megalin downregulation in IgA nephropathy (13, 16). In a subsequent cohort study, lower renal megalin expression was associated with a higher risk of CKD progression, whereas higher renal miR-148b expression identified patients at increased risk of progression, defined as ESRD or an eGFR decline of at least 40% (13). Plasma miR-148b-3p has also been reported to be elevated in diabetes and prediabetes, with associations with fasting glucose and selected lipid indices (14). Taken together, these observations suggest that miR-148b-3p is not an isolated molecular signal but part of a broader CKD-related and metabolism-related miRNA remodeling process.

Against this background, our finding that plasma miR-148b-3p was elevated in CKD and inversely associated with eGFR supports the view that miR-148b-3p reflects CKD-related molecular remodeling. The association with renal function was one of the most consistent findings in our study, indicating that miR-148b-3p increased as kidney function declined. Clinically, miR-148b-3p was associated with hypertension and diabetes mellitus, 2 of the most common CKD-associated comorbidities and major drivers of ongoing renal injury, which may suggest that higher miR-148b-3p levels indicate greater kidney damage in these patients (14). The association with diabetes appears biologically plausible, as plasma miR-148b-3p has been reported to be elevated in diabetes and prediabetes and to correlate with fasting glucose and selected lipid indices, supporting a link with metabolic dysregulation (14). By contrast, direct evidence linking miR-148b-3p specifically to hypertension remains limited; a more cautious interpretation is that the association observed in our cohort reflects the broader hypertensive CKD milieu, in which sustained hemodynamic stress, renin-angiotensin system activation, oxidative stress, and vascular inflammation promote progressive kidney and vascular injury and may secondarily shape circulating miRNA profiles (17, 18). Conversely, elevated miR-148b-3p may further aggravate vascular endothelial injury, thereby favoring a prohypertensive vascular state and metabolic dysfunction, including insulin resistance (19, 20). Although HDL-C was lower in patients with higher miR-148b-3p expression, there is currently no direct evidence linking HDL-C to miR-148b-3p. A more plausible explanation is that both changes reflect the CKD milieu, where chronic inflammation, oxidative stress, and uremic metabolic disturbance contribute to HDL dysfunction while simultaneously driving miR-148b-3p elevation (20).

Our results further showed that plasma miR-148b-3p increased progressively across tertiles of plaque burden and remained independently associated with Crouse score after multivariable adjustment, suggesting that it may capture a component of CKD-related vascular injury not reflected by routine clinical variables alone. Plasma miR-148b-3p levels were markedly right-skewed (skewness, 3.59; Shapiro–Wilk *P* < 0.001) (Supplementary Table S7). Log2 transformation substantially reduced skewness (0.89), although the distribution remained nonnormal (*P* < 0.001). In the fully adjusted eGFR model, residual normality and homoscedasticity were acceptable for both the raw and log2-transformed miR-148b-3p specifications. Model specification was borderline for the raw exposure (Ramsey RESET *P* = 0.050) but acceptable after log2 transformation (*P* = 0.564) (Supplementary Table S8). Residuals from the Crouse score models remained nonnormal, despite no evidence of heteroscedasticity or model misspecification; these findings should therefore be interpreted cautiously. Mechanistically, altered miR-148b-3p signaling may reflect endothelial-related vascular stress by disrupting endothelial homeostasis, impairing adaptive responses to inflammatory and oxidative stress, and promoting extracellular matrix remodeling within the vascular wall (11, 12, 19, 21). These changes may weaken endothelial integrity, favor a proinflammatory and pro-remodeling vascular phenotype, and thereby facilitate plaque progression. Accordingly, miR-148b-3p may represent not only a marker of CKD-related inflammatory and metabolic stress, but also a potential marker of molecular processes associated with endothelial dysfunction and atherosclerotic progression. The ROC findings should be interpreted as exploratory. Plasma miR-148b-3p showed limited discrimination for carotid plaque in the non-CKD group and moderate discrimination in the CKD subgroup. Although bootstrap resampling suggested internal stability of the AUC estimate, bootstrap validation does not assess external transportability and cannot fully eliminate optimism arising from model development and evaluation within the same cohort. In addition, the CKD subgroup included only 90 participants, of whom 16 were free of carotid plaque, resulting in an imbalanced outcome distribution and limited effective sample size for predictive modeling. Although addition of miR-148b-3p improved several discrimination and reclassification indices, the bootstrap confidence interval for ΔAUC included zero, and these estimates may be sensitive to overfitting. Therefore, the present findings do not establish miR-148b-3p as a clinically validated diagnostic biomarker for carotid plaque.

Integrative bioinformatic analyses further supported the biological plausibility of this framework. Predicted miR-148b-3p targets were enriched in pathways related to endothelial function and atherogenesis, including angiogenesis, vasculature development, cell adhesion, PI3K-Akt signaling, and TGF-β signaling. Single-cell analysis showed reduced expression of endothelial homeostasis–related genes in symptomatic vs. asymptomatic plaques, consistent with more advanced endothelial dysfunction. Integration of these data with our CKD endothelial transcriptomic dataset identified four shared candidate genes—MRPS25, ADAMTS5, MMP15, and RAB12—implicated in mitochondrial translation and oxidative phosphorylation, extracellular matrix remodeling, angiogenesis, and autophagic-lysosomal trafficking (22–31). Collectively, these findings suggest that elevated miR-148b-3p may be associated with a molecular pattern involving impaired endothelial metabolic adaptation, matrix remodeling, and vascular stability. However, direct regulation of these targets by miR-148b-3p and their functional contribution to plaque burden remain to be established.

The present study has several limitations. First, its single-center, cross-sectional design precludes causal inference and assessment of temporal relationships. It therefore remains unclear whether elevated plasma miR-148b-3p precedes renal function decline or plaque progression, or instead reflects established CKD-related vascular injury. Second, all participants had established CAD. Although this enabled evaluation of CKD-related differences within a high-risk ASCVD population, it limits generalizability to individuals without CAD and to community-based CKD populations. Third, the sample size was modest, particularly for subgroup ROC and incremental predictive analyses. Although bootstrap analyses were performed, the diagnostic and predictive performance of miR-148b-3p was not validated in an independent cohort; accordingly, these biomarker findings should be considered preliminary. Fourth, CKD is etiologically and clinically heterogeneous. Although analyses across eGFR categories were performed, the numbers of participants with advanced kidney dysfunction were small, and quantitative albuminuria data, underlying renal diagnosis, CKD duration, and longitudinal changes in renal function were unavailable or incomplete. These limitations precluded robust stratification by CKD severity, albuminuria burden, or etiology. Fifth, the mechanistic analyses relied on target prediction, public single-cell transcriptomic data, and a CKD endothelial RNA-sequencing reference dataset. Differences in species, vascular beds, disease models, and sequencing platforms may have introduced biological and technical heterogeneity. Sixth, the fully adjusted logistic models included a relatively large number of covariates for the available sample size. Conservative events-per-variable values were low in the overall, CKD, and non-CKD models (4.38, 1.07, and 3.07, respectively), particularly because only 16 patients with CKD were free of carotid plaque. This imbalance may have increased the risk of overfitting, unstable coefficients, and imprecise estimates; therefore, the subgroup and incremental predictive analyses should be considered exploratory and require validation in larger cohorts with more balanced outcome distributions (Supplementary Table S9). Finally, direct functional validation was not performed. Whether miR-148b-3p directly regulates the identified candidate targets and whether these targets contribute functionally to endothelial injury or carotid plaque burden remain to be established.

In conclusion, among patients with established CAD, plasma miR-148b-3p was elevated in those with CKD and was independently associated with lower eGFR and greater carotid plaque burden. Exploratory ROC and incremental predictive analyses suggested that miR-148b-3p may provide information relevant to plaque assessment in the CKD subgroup; however, these analyses do not establish clinical diagnostic utility because of the modest subgroup size, imbalanced outcome distribution, potential overfitting, and absence of external validation. Integrative transcriptomic analyses identified several endothelial- and vascular-relevant candidate targets, including MRPS25, ADAMTS5, MMP15, and RAB12, but these findings remain hypothesis-generating. Larger prospective multicenter studies with standardized comparison against established renal and inflammatory biomarkers and independent external validation are required to determine whether plasma miR-148b-3p provides clinically meaningful incremental information.

## Data Availability

The datasets presented in this study can be found in online repositories. The names of the repository/repositories and accession number(s) can be found in the article/Supplementary Material.
