# Supplementary material for: Plasma miR-148b-3p is associated with CKD-related renal dysfunction and carotid plaque burden in patients with established coronary artery disease
Source: Front Cardiovasc Med. 2026 Aug 12;13:1908726. doi: 10.3389/fcvm.2026.1908726 (PMC13507669; doi:10.3389/fcvm.2026.1908726)
Supplement: Supplementary file 1 [file Datasheet1.docx]

**Supplementary Table S1. Primer sequences used for plasma miR-148b-3p quantitative PCR**

| **miR-148b-3p RT** | CTCAACTGGTGTCGTGGAGTCGGCAATTCAGTTGAGACAAAGTT |
| --- | --- |
| **miR-148b-3p F** | ACACTCCAGCTGGGTCAGTGCATCACAGAA |
| **miR-cel39-3pRT** | CTCAACTGGTGTCGTGGAGTCGGCAATTCAGTTGAGCAAGCTGA |
| **miR-cel39-3pF** | ACACTCCAGCTGGGATCACCGGGTGTAAAT |
| **miR-R-total** | CTGGTGTCGTGGAGTCGG |

**Supplementary Table S2. Variance inflation factor analysis for the fully adjusted models**

| **Variable** | **VIF** |
| --- | --- |
| Plasma miR-148b-3p level | 1.13 |
| Age | 1.30 |
| Male sex | 1.53 |
| BMI | 1.14 |
| Hypertension | 1.24 |
| Diabetes mellitus | 1.11 |
| Smoking history | 1.48 |
| TC | 6.57 |
| TG | 1.47 |
| HDL-C | 1.49 |
| LDL-C | 6.62 |
| ACEI use | 1.16 |
| ARB use | 1.27 |
| β-blocker use | 1.31 |
| Statin use | 1.26 |

**Supplementary Table S3.** **Bootstrap validation of the discriminative performance of plasma miR-148b-3p for carotid plaque detection**

| **Subgroup** | **n** | **Apparent AUC** | **Bootstrap mean AUC** | **Bootstrap 95% CI** | **Bootstrap resamples** |
| --- | --- | --- | --- | --- | --- |
| Non-CKD | 100 | 0.524 | 0.523 | 0.411–0.638 | 1000 |
| CKD | 90 | 0.780 | 0.782 | 0.667–0.885 | 1000 |

**Supplementary Table S4. Incremental predictive value of plasma miR-148b-3p for carotid plaque beyond clinical variables in the CKD subgroup**

| **Cohort** | **n** | **Clinical AUC** | **Clinical + miR AUC** | **ΔAUC** | **DeLong P** | **LRT P** | **Continuous NRI** | **IDI** | **Bootstrap ΔAUC,**  **mean (95% CI)** | **Bootstrap continuous NRI,**  **mean (95% CI)** | **Bootstrap IDI,**  **mean (95% CI)** |
| --- | --- | --- | --- | --- | --- | --- | --- | --- | --- | --- | --- |
| CKD | 90 | 0.696 | 0.794 | 0.098 | 0.019 | 0.001 | 0.682 | 0.114 | 0.065 (−0.001, 0.171) | 0.818 (0.088, 1.615) | 0.141 (0.008, 0.449) |

| **Analysis domain** | **Cohort** | **Outcome / association / model** | **n** | **Events / nonevents** | **Observed effect size** | **Achieved power** | **Required sample size or event count for 80% power** |
| --- | --- | --- | --- | --- | --- | --- | --- |
| Correlation | Overall | miR-148b-3p vs eGFR | 190 | — | Spearman r=−0.310 | 0.992 | Required n=80 |
| Correlation | CKD | miR-148b-3p vs eGFR | 90 | — | Spearman r=−0.460 | 0.996 | Required n=35 |
| Correlation | Overall | miR-148b-3p vs Cys-C | 190 | — | Spearman r=0.219 | 0.861 | Required n=162 |
| Correlation | CKD | miR-148b-3p vs Cys-C | 90 | — | Spearman r=0.361 | 0.941 | Required n=59 |

**Supplementary Table S5. Post-hoc detectable-effect assessment for correlations between plasma miR-148b-3p and renal-function indices**

| **G_stage** | **n** | **eGFR_mean_SD** | **miR148b_mean_SD** | **rho** | **P_value** |
| --- | --- | --- | --- | --- | --- |
| G1 (>=90) | 102 | 145.19 ± 29.34 | 2.69 ± 3.86 | -0.147 | 0.141 |
| G2 (60-89) | 51 | 77.94 ± 9.85 | 4.61 ± 8.54 | -0.368 | 0.008 |
| G3 (30-59) | 30 | 48.39 ± 6.39 | 12.60 ± 18.21* | -0.066 | 0.729 |
| G4 (15-29) | 6 | 21.35 ± 4.23 | 22.42 ± 19.58* | 0.257 | 0.623 |
| G5 (<15) | 1 | 14.04 ± NA | 10.03 ± NA |  |  |

**Supplementary Table S6. Plasma miR-148b-3p Levels and Their Associations With eGFR Across eGFR Categories**

Values are presented as mean±SD. Participants were categorized according to eGFR as follows: G1, ≥90; G2, 60–89; G3, 30–59; G4, 15–29; and G5, <15 mL/min/1.73 m². Correlations between plasma miR-148b-3p levels and eGFR within each category were assessed using Spearman rank correlation analysis. The reported P values correspond to the within-category Spearman correlations. Overall differences in plasma miR-148b-3p levels across eGFR categories were evaluated using the Kruskal–Wallis test, followed by Dunn post hoc comparisons with G1 as the reference. *P<0.05 versus G1 group. The correlation coefficient and P value were not estimated for G5 because this category included only 1 participant.

**Supplementary Table S7. Distributional Characteristics of Plasma miR-148b-3p Before and After Log2 Transformation**

| **Variable** | **n** | **Mean_SD** | **Median_IQR** | **Skewness** | **Excess_kurtosis** | **Shapiro_W** | **Shapiro_P_value** |
| --- | --- | --- | --- | --- | --- | --- | --- |
| Raw plasma miR-148b-3p (2^−ΔΔCt) | 190 | 5.43 ± 10.49 | 1.81 [0.42, 5.37] | 3.590238 | 14.23016 | 0.523518 | 1.79E-22 |
| log2(plasma miR-148b-3p + 1) | 190 | 1.75 ± 1.47 | 1.49 [0.51, 2.67] | 0.88624 | 0.22137 | 0.914015 | 4.38E-09 |

Plasma miR-148b-3p was quantified as relative expression using the 2^−ΔΔCt method. The transformed variable was calculated as log2(miR-148b-3p+1). Skewness and excess kurtosis were used to characterize distributional asymmetry and tail behavior. Departure from normality was assessed using the Shapiro–Wilk test, with P<0.05 indicating a statistically significant deviation from a normal distribution.

**Supplementary Table S8. Residual Diagnostics for Fully Adjusted Linear Regression Models Using Raw and Log2-Transformed Plasma miR-148b-3p**

| **Outcome** | **Exposure_scale** | **Shapiro P** | **Breusch Pagan P** | **Ramsey RESET P** |
| --- | --- | --- | --- | --- |
| eGFR | Raw miR-148b-3p | 0.348 | 0.123 | 0.050 |
| Crouse score | Raw miR-148b-3p | 3.378E-18 | 0.601 | 0.203 |
| eGFR | log2(miR-148b-3p + 1) | 0.298 | 0.127 | 0.564 |
| Crouse score | log2(miR-148b-3p + 1) | 3.742E-18 | 0.582 | 0.185 |

Plasma miR-148b-3p was modeled using either the raw 2^−ΔΔCt value or log2(miR-148b-3p+1). Residual normality was evaluated using the Shapiro–Wilk test, homoscedasticity using the Breusch–Pagan test, and model functional form using the Ramsey RESET test. For each test, P<0.05 indicates evidence of residual nonnormality, heteroscedasticity, or potential model misspecification, respectively.

**Supplementary Table S9. Residual Degrees of Freedom and Events per Variable for Logistic Regression Models of Carotid Plaque**

| **Outcome** | **Model** | **n** | **Events** | **Nonevents** | **Minority class, n** | **Predictors, n** | **Residual df** | **Event EPV** | **Conservative EPV** |
| --- | --- | --- | --- | --- | --- | --- | --- | --- | --- |
| Carotid plaque | overall Model 3 | 190 | 120 | 70 | 70 | 16 | 173 | 7.50 | **4.38** |
| Carotid plaque | CKD subgroup Model 3 | 90 | 74 | 16 | 16 | 15 | 74 | 4.93 | **1.07** |
| Carotid plaque | non-CKD subgroup Model 3 | 100 | 46 | 54 | 46 | 15 | 84 | 3.07 | **3.07** |

Residual degrees of freedom were calculated as n minus the number of predictors minus 1. Event EPV was calculated as the number of participants with carotid plaque divided by the number of predictors. Conservative EPV was calculated using the smaller of the event and nonevent counts divided by the number of predictors. An EPV of 10 is commonly used as a rule of thumb for assessing model stability but should not be considered an absolute validity threshold.

**Supplementary Table S10. Bootstrap-Validated Discrimination of Cystatin C for Carotid Plaque in CKD and Non-CKD Subgroups**

| **Subgroup** | **n** | **Apparent AUC** | **Bootstrap mean AUC** | **Bootstrap 95% CI** | **Bootstrap resamples** |
| --- | --- | --- | --- | --- | --- |
| Non-CKD | 100 | 0.554 | 0.552 | 0.441–0.661 | 1000 |
| CKD | 90 | 0.638 | 0.639 | 0.483–0.781 | 1000 |

Apparent AUCs were calculated from the original subgroup data. Bootstrap mean AUCs and 95% confidence intervals were estimated using 1,000 resamples with replacement. The 95% confidence intervals were derived using the percentile method. Carotid plaque status was treated as the binary outcome, with higher cystatin C levels indicating a greater probability of plaque presence.

**Figure S1. Association between plasma miR-148b-3p levels and cystatin C in patients with established CAD**

Scatter plot showing the association between plasma miR-148b-3p levels and serum cystatin C (Cys-C) in the overall study population. Circles represent patients without chronic kidney disease (non-CKD), and triangles represent patients with CKD. Each symbol represents one participant. The solid red line indicates the fitted trend, and the shaded area represents the 95% confidence interval. Plasma miR-148b-3p levels were positively correlated with Cys-C (Spearman r=0.22, P=0.002).

**Figure S2. ROC Curves of Cystatin C for Carotid Plaque in Non-CKD and CKD Subgroups**

**B**

**A**

(A) In the non-CKD subgroup, cystatin C yielded an AUC of 0.554, with an optimal cutoff of 0.555 mg/L, a sensitivity of 97.8%, and a specificity of 16.7%. (B) In the CKD subgroup, cystatin C yielded an AUC of 0.638, with an optimal cutoff of 1.600 mg/L, a sensitivity of 47.3%, and a specificity of 87.5%.
